# Flow cytometry for extracellular vesicle characterization in COVID-19 and post-acute sequelae of SARS-CoV-2 infection

**DOI:** 10.20517/evcna.2024.20

**Published:** 2024-08-09

**Authors:** Marialaura Fanelli, Vita Petrone, Rossella Chirico, Claudia Maria Radu, Antonella Minutolo, Claudia Matteucci

**Affiliations:** ^1^Department of Experimental Medicine, University of Rome Tor Vergata, Rome 00133, Italy.; ^2^Department of Medicine - DIMED, Thrombotic and Hemorrhagic Diseases Unit, University of Padua, Padua 35128 Italy.; ^#^Authors contributed equally.

**Keywords:** Multidistrict infection, multiparametric analysis, biomarkers, flow cytometry, COVID-19, SARS-CoV-2, PASC, Long COVID

## Abstract

Infection with SARS-CoV-2, the virus responsible for COVID-19 diseases, can impact different tissues and induce significant cellular alterations. The production of extracellular vesicles (EVs), which are physiologically involved in cell communication, is also altered during COVID-19, along with the dysfunction of cytoplasmic organelles. Since circulating EVs reflect the state of their cells of origin, they represent valuable tools for monitoring pathological conditions. Despite challenges in detecting EVs due to their size and specific cellular compartment origin using different methodologies, flow cytometry has proven to be an effective method for assessing the role of EVs in COVID-19. This review summarizes the involvement of plasmatic EVs in COVID-19 patients and individuals with Long COVID (LC) affected by post-acute sequelae of SARS-CoV-2 infection (PASC), highlighting their dual role in exerting both pro- and antiviral effects. We also emphasize how flow cytometry, with its multiparametric approach, can be employed to characterize circulating EVs, particularly in infectious diseases such as COVID-19, and suggest their potential role in chronic impairments during post-infection.

## INTRODUCTION

The capacity and necessity to communicate are fundamental to normal cell functioning. Cell activity and survival are closely linked to the reception and processing of information from itself and the external environment. Cells can communicate directly with each other through a series of chemical and mechanical signals, enabling the specialization of groups of cells that can form tissues such as muscles, blood, and brain tissue^[1]^. The ability of cells to communicate is also mediated through the release of vesicles into the extracellular environment, commonly called extracellular vesicles (EVs). EVs can reach target cells through biological fluid and transmit their cargo molecules, determining the fate of the recipient cell through endocrine, paracrine, or autocrine signaling^[2]^. They also play the role of removing unwanted molecular material or cellular waste^[3]^.

EVs are cell-derived vesicles characterized by a lipid bilayer and cannot replicate with a size between 30 nm and 5 µm; they are secreted into the extracellular space by various cells under physiological and pathological conditions. EVs are primarily distinguished into three main categories: exosomes, which have a diameter of less than 200 nm and originate from endosomal cellular compartments within the cell; ectosomes [also called microvesicles (MVs)], which originate from the cellular membrane surface; and apoptotic bodies, which are derived from cells undergoing programmed cell death. EVs are further classified into two main groups based on size: small EVs (with a diameter < 200 nm) and large EVs (with a diameter > 200 nm) diameter. This classification is due to the overlapping size of exosomes and MVs^[4,5]^. The term “exosomes” specifically refers to vesicles originating from the endosomal system and are generally less than 200 nm.

Ectosomes are vesicles that arise from the plasma membrane and can vary in size, including sizes similar to those of exosomes^[5]^. The functional impact of EVs on recipient cells and their cellular origin contribute to their heterogeneity^[6-8]^.

The lipid bilayer of EVs is characterized by the presence of membrane proteins including tetraspanins such as CD9, CD63, CD81 and CD82, cell adhesion-related proteins, MVB-related proteins (TSG101, ALIX and Rab proteins), antigen presentation-related proteins (class I and II of the major histocompatibility complex), peptide death receptors [fasL and tumor necrosis factor (TNF) - apoptosis inducing ligand], and iron transport proteins (transferrin receptor)^[9]^.

The formation of EVs is mediated by the presence of various fatty acids and lipids belonging to plasma membranes, including ceramide, cholesterol, sphingomyelin, and phosphatidylserine^[10-13]^. The presence of EVs has been demonstrated in different biological fluids [blood, urine, milk, nasal secretions, amniotic fluid, cerebrospinal fluid (CSF), and broncho-alveolar lavage fluid (BALF)], highlighting their physiological ability as transducers and carriers^[14,15]^.

## THE IMPACT OF SARS-COV-2 INFECTION ON CELLS: FROM CELLULAR ORGANELLES ALTERATION TO EVS PRODUCTION

**Figure 1:**
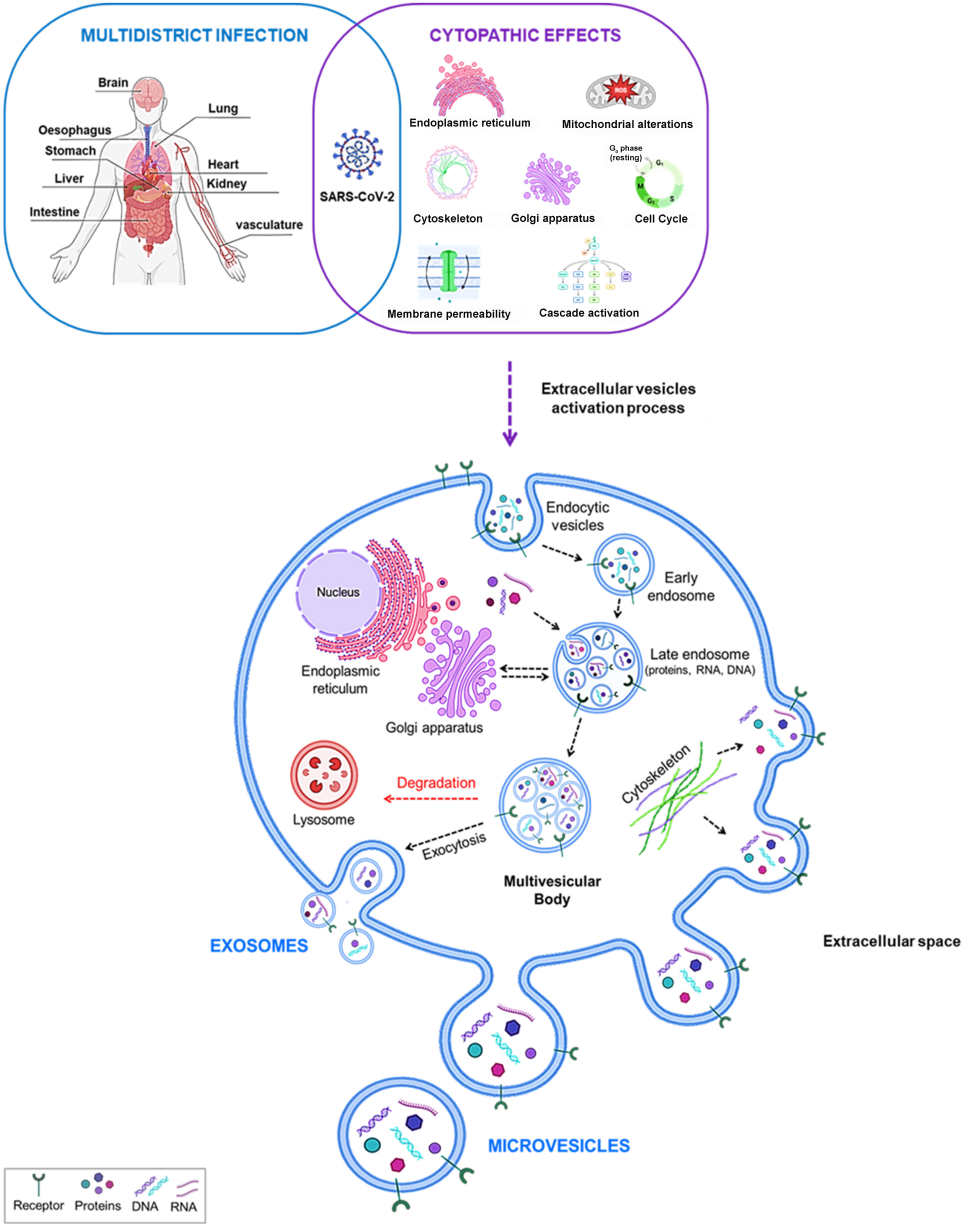
The release of EVs as a result of the multilevel impairments resulting from SARS-CoV-2 infection. Created with BioRender.com. EVs: Extracellular vesicles.

Due to external insults such as infectious pathogens (viruses, bacteria, fungi)^[16]^, drugs, physical agents^[17,18]^, or tumorigenesis^[19]^, cells modify their phenotype at the membrane surface and intracellular levels. In particular, during viral infections, host cells undergo major changes in their morphology and molecular composition, deviating from their normal physiological state. Virus infection results in various cytopathic effects (CPE), such as rounding of the infected cell, increased fusion processes with adjacent cells (also known as syncytia), and the formation of nuclear or cytoplasmic inclusion bodies. Other important effects involving the alteration of normal functions include the activation of signal cascades, transcription activation or inhibition, changes in cell membrane permeability, metabolism and the cell cycle^[20,21]^, and known genotoxic effects^[22]^ [Figure 1].

The consequences at the multidistrict level of SARS-CoV-2 infection have been extensively described^[23]^, demonstrating how virus replication has an impact in the human body.

The ability of SARS-CoV-2 to induce changes in cell and/or tissue physiology is closely related to the specific cell type involved^[24]^.

The widespread expression of ACE2, a receptor that SARS-CoV-2 uses to enter cells, in multiple organs facilitates the spread of the virus. Therefore, SARS-CoV-2 can induce neurological, cardiac, pulmonary, gastrointestinal, hepatic, renal, and vascular impairment^[25]^. More specifically, the Spike protein, a viral glycoprotein, is cleaved by TMPRSS2, which triggers viral activation and is a crucial host factor for SARS-CoV-2 pathogenicity. This mechanism is similar to that observed in other coronaviruses, such as SARS-CoV and influenza viruses like H1N1 influenza^[26]^. This makes TMPRSS2 an attractive pharmacological target for impeding viral entry^[27]^. Additionally, other membrane proteins participate in the internalization of SARS-CoV-2. One such protein is Siglec-1/CD169, which is known to bind and internalize HIV at the cellular level^[28,29]^. Siglec-1/CD169 interacts with sialylated gangliosides on the membranes of SARS-CoV-2 variants. Research has shown that blocking Siglec-1 on monocyte-derived dendritic cells reduces the viral transfer of SARS-CoV-2 to target cells^[30]^. Another important protein is neuropilin 1^[31]^, a surface protein with various functions that binds to VEGF and semaphorin^[32]^ and has an important role in the development of neurons and the cardiovascular system. During infection, NRP1-promoted virus entry is thought to facilitate the infection of cells with low levels of ACE2 expression, such as olfactory endothelial cells^[33]^, possibly aiding the separation of S1 and S2 subunits of the Spike protein^[34]^. Indeed, NRP1-mediated entry could have important neurological implications, especially in the olfactory-related region of the CNS, contributing to the neurological symptoms associated with SARS-CoV-2^[35]^. Once inside the host cell, SARS-CoV-2 disrupts the cellular microenvironment by altering cellular structures and organelles [Figure 1].

In particular, SARS-CoV-2 has been shown to contribute to the alteration of mitochondrial homeostasis^[36]^. Shang *et al.* demonstrated the presence of SARS-CoV-2 viral RNA in mitochondria, causing increased reactive oxygen species (ROS) release and inhibition of mitophagy at an early stage^[37]^.

Among its numerous effects, SARS-CoV-2 has also been shown to significantly impact the endoplasmic reticulum^[38]^, Golgi apparatus^[39]^, and cytoskeleton^[40]^, which are all structures physiologically involved in the biogenesis and secretion of EVs^[41,42]^. As with many other viruses, vesicular structures appear to play a key role in SARS-CoV-2 infection at the morphological level, participating in virus assembly, egress from infected cells, and spread to other cellular targets.

EVs can have either beneficial or detrimental effects on SARS-CoV-2 infection, depending on their load. Virus-infected cells show increased production of EVs, which transfer viral nucleic acids, proteins, entire virions, or viral entry receptors to healthy cells. This not only facilitates the spread of the virus but also influences the immune responses and susceptibility of target cells to infection^[43]^.

Considering the important damage generated by SARS-CoV-2 at the cellular, systemic, and immunological levels, EVs represent another element to be considered due to their ability to transport material (RNA, DNA, proteins) from the infected or non-infected secreting cell^[15,43]^. Indeed, EVs generated from virus-infected cells can act as carriers of virulence factors, including viral proteins and genetic material, sustaining the infection itself^[44]^ [Figure 1].

SARS-CoV-2 can generate multidistrict infection, resulting in alterations in different organs. At the cellular compartment, SARS-CoV-2 induces several cytopathic effects (alteration at the level of the Endoplasmic reticulum and Golgi apparatus, mitochondrial, cytoskeleton, cell cycle, and membrane permeability), some of which are strictly involved in EVs biogenesis and release.

Based on this evidence, characterizing patient responses by exploiting the molecular profiling of EVs to find biomarkers of disease progression may be of great importance in finding therapeutic targets to mitigate the effects of SARS-CoV-2 infection. For instance, as reported by the proteomic analysis of EV load from a cohort of COVID-19 patients, three clusters with specific functions (exRNAs related to liver damage, EVs proteins related to antiviral response, and coagulation-related markers) were clearly defined, indicating that EVs may reflect host-specific responses to infection, as well as disease progression^[45]^.

## STATE OF THE ART: THE EVS INVOLVEMENT IN COVID-19 AND POST-ACUTE SEQUELAE OF SARS-COV-2 INFECTION

COVID-19, caused by the SARS-CoV-2 virus infection, is a disease with a multidistrict impact. It has been widely documented that it can lead to failures in the respiratory, immune, cardiovascular, and neurological systems^[46,47]^.

In many cases, resolving the infection does not fully address the major impairments that affect various parts of the body. Complex symptoms can persist for four weeks or more after the initial infection and are defined by the Centers for Disease Control and Prevention (CDC) as “post-COVID conditions” (PCC) or post-acute sequelae of SARS-CoV-2 infection (PASC). These conditions are characterized by social, physical, and psychological impairments^[48-50]^.

The symptoms of PASC are very heterogeneous and could emerge during the acute phase of the infection and continue after its resolution, or they may develop as a consequence of COVID-19^[51-53]^.

In this complex scenario, this review aims to describe and highlight the potential role of EVs in both COVID-19 and PASC. Due to their peculiar characteristics (small size, varied membrane markers, and ability to carry different materials), detecting EVs is difficult. Currently, there is no highly accurate and standardized method that can simultaneously assess all these features.

**Figure 2:**
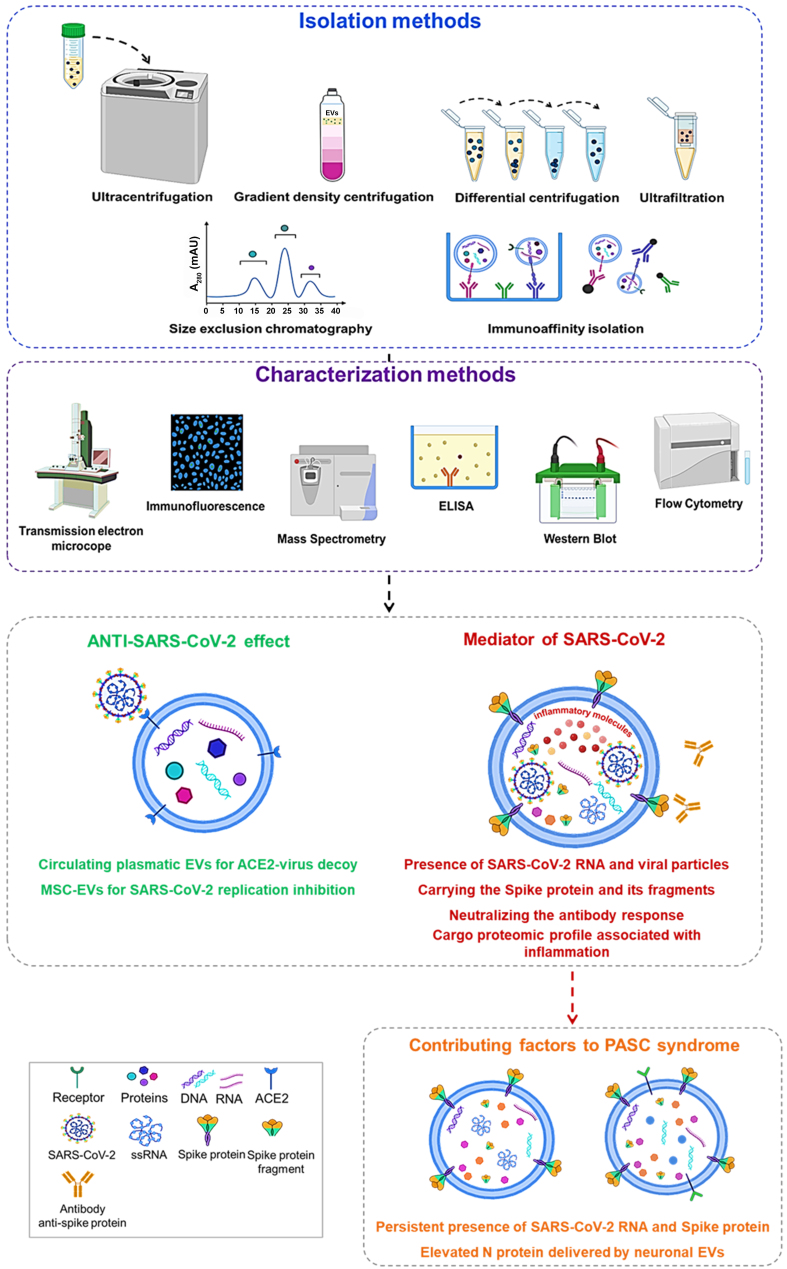
The potential role of EVs in COVID-19 and PASC: from isolation methods to characterization. Created with BioRender.com. EVs: Extracellular vesicles; PASC: post-acute sequelae of SARS-CoV-2 infection.

In the COVID-19 context, different methods have been used for the detection and characterization of EVs. As reported in a recent work, different approaches such as ultracentrifugation, density gradient centrifugation, size exclusion chromatography, immunoaffinity isolation methods, microfluidics-based methods, and reduced solubility approaches have been developed to better isolate EVs and especially to establish standard guidelines^[54,55]^. Various methodologies such as transmission electron microscope, immunofluorescence, mass spectrometry, enzyme-linked immunosorbent assay (ELISA), western blot, and flow cytometry have been used for EV characterization [Figure 2].

In the context of COVID-19, EVs were studied and evaluated in terms of their potential role as mediators of infection and associated pathological disorders.

In recent work, the exosomes recovered from the plasma of COVID-19 patients were demonstrated to carry fragments derived from the spike protein, through chip-based immunofluorescence. In addition, it was shown by mass spectrometry that the exosome proteome from patients with mild conditions correlated with adequate immune system activity, while that of severe patients with increased and chronic inflammation^[56]^. Moreover, EVs have been found to incorporate the Spike protein of SARS-CoV-2 and serve as a target for antibodies derived from the serum of COVID-19 convalescent patients, which can interfere with their ability to block virus entry^[57]^. Berry *et al.* demonstrated that EVs present in mucus produced by human nasal epithelial cells express receptor ACE2 and the activated protease TMPRSS2 and have been shown to trigger Spike prefusion by promoting viral tropism^[58]^.

In the context of infection, Xia *et al.* demonstrated by transmission electron microscopy analysis that SARS-CoV-2-induced EVs contain large amounts of live viral particles. Notably, the SARS-CoV-2 virus carried in vesicles was resistant to neutralizing antibodies and was able to reinfect cells independently of receptors and signaling cofactors^[59]^. Interestingly, the quantitative proteomics confirmed that the Spike protein could have the ability to interact with the machinery involved in EV biogenesis^[60,61]^.

However, due to their similar sizes, completely separating EVs from viral particles is very challenging. Although there is considerable heterogeneity between EVs and viruses, numerous isolation methods are sometimes used for both. McNamara *et al.* define several techniques for the isolation of EVs and viruses, highlighting the advantages and limitations of each method. Notably, their work highlights the peculiarity of the flow cytometry method (nanoFACS), which can separate EVs from viruses based on specific markers^[62]^. Furthermore, it is important to note that not all viral inactivation methods, which are used to safely manipulate samples, are compatible with the protocols for EV isolates contaminated with SARS-CoV-2^[63]^. Therefore, any infection experiment using EV isolates from virus-containing samples must be interpreted with caution.

In contrast, the EVs-mediated antiviral effects were investigated. In particular, mesenchymal stem cell-derived (MSC) EVs have shown the ability to suppress viral replication and attenuate virion release and production in Calu-3 cells infected with SARS-CoV-2. Furthermore, this mechanism does not interfere with the virus entry process^[64]^. Furthermore, miRNAs present in EVs block SARS-CoV-2 through direct binding to the 3’ UTR portion, resulting in translational repression and the ability to prevent cytokine storms and counteract inflammatory responses and immunopathogenesis^[65]^. Furthermore, in hospitalized patients with severe COVID-19, intravenous administration of MSC-SEV significantly alleviated hypoxia, immune recovery, and cytokine storm, without causing adverse reactions^[66]^.

In COVID-19 patients, an increase in circulating EVs expressing ACE2, and their ability to neutralize SARS-CoV-2 have been demonstrated. This antiviral mechanism is explained by competition between the ACE2 receptors present in the vesicles (ACE2-positive EVs) and those on the cells^[67]^.

EVs could also contribute to the progression of COVID-19. Proteomic analysis of patient-derived exosomes has identified several molecules involved in the main mechanisms of COVID-19-related tissue damage and multiple organ dysfunction, such as immune response, inflammation, and coagulation, including the presence of SARS-CoV-2 RNA^[68]^. In connection with the coagulation abnormalities observed in COVID-19 patients^[69]^, Rosell *et al.* reported the presence of plasmatic EVs expressing tissue factor (TF), the main activator of the coagulation cascade; furthermore, the activity of TF-bearing EVs was linked to disease severity and mortality, and showed a correlation with D-Dimer levels^[70]^.

Multidistrict impairments resulting from COVID-19 progression often persist even after the infection has resolved, leading to a condition known as Long COVID (LC). Individuals with LC are affected by PASC, which are often associated with severe neurological disorders.

In this context, there is limited information regarding the involvement of EVs involvement in PASC syndrome.

A recent study analyzed the levels of Spike protein and viral RNA contained in circulating EVs of hospitalized patients with acute COVID-19, as well as patients with and without PASC symptoms. The study found that 30% of the PASC individuals were positive for both Spike and viral RNA, while none of the individuals without symptoms tested positive^[71]^.

In individuals with PASC, analysis using ELISA method revealed high expression of S1 and nucleocapsid (N) proteins of SARS-CoV-2 in circulating neuron- and astrocyte-derived EVs from plasma. Notably, higher levels of N proteins were associated with neuropsychiatric symptoms^[72]^.

In other research, neuronal-enriched EVs (nEVs), characterized by the expression of the neuronal marker L1CAM, were detected in the plasma of PASC individuals. Compared to healthy controls, nEVs from PASC individuals showed elevated levels of protein markers associated with neuronal dysfunction. This suggests ongoing peripheral neuroinflammation following COVID-19, which may influence neurological sequelae by altering nEV proteins^[73]^ [Figure 2].

This evidence underscores the dual role of EVs during SARS-CoV-2 infection. They can function both as vehicles for promoting and spreading the virus and as elements that can counteract viral mechanisms. In the complex scenario of COVID-19 and its post-infection sequelae, several studies suggest that EVs are capable of transmitting pro-inflammatory and immune-stimulatory signals from various parts of the body, including the nervous system.

Different isolation and characterization methods have enabled the evaluation of the possible roles of EVs (MVs and exosomes). The anti- and pro-infection roles of EVs were highlighted based on the presence of endogenous (ACE2 receptor) and SARS-CoV-2-derived materials (RNA and protein).

## FLOW CYTOMETRY APPROACH DECIPHERING COVID-19 AND PASC: FROM CELLS TO EVS

In recent years, the quest to uncover and understand the mechanisms underlying complex diseases has led researchers to adopt a multiparametric approach. The integration and refinement of various methods have improved the study of different parameters that contribute to the development and resolution of these pathologies^[74]^. Among the many approaches, flow cytometry stands out as a highly reliable and sensitive multiparametric method. It is ideal for a detailed analysis of single cells or particles in suspension. Flow cytometry provides both qualitative and quantitative assessments of multiple characteristics, enabling the discrimination of size, complexity, and the expression of extra- and intracellular molecules. Therefore, it has been applied to the study of various complex diseases, including cancer, neurodegenerative disorders, infectious diseases, and cardiovascular conditions^[75]^.

Flow cytometry has also been used to analyze single EVs. The primary challenges in identifying and characterizing EVs include their small size and high heterogeneity of biomarkers, as well as low refractive index. However, advancements in nanotechnology have led to the development of improved tools for analyzing small particles, which enhances the study of EVs from various sources (animal, plant, and microbial) and highlights their heterogeneity^[76]^.

Nano-flow cytometry (nFCM) is an emerging technique for nanoparticle characterization and can detect particles as small as 30 nm. The high sensitivity of nFCM’s lateral scattering detection enables multi-parameter quantitative analysis of single particles based on their distribution and concentration^[77]^.

Through flow cytometry, the heterogeneity of EVs can be analyzed through phenotyping and quantification^[78,79]^.

### Cells characterization

To study and characterize the immune response to COVID-19, flow cytometry was employed to phenotype T cells, B cells, NK cells, dendritic cells, and monocyte subpopulations, evaluating their differentiation and activation status^[80-83]^. This approach revealed several alterations in both innate and adaptive immunity, facilitating the identification of predictive markers associated with disease stage and progression^[84-86]^.

The ability of SARS-CoV-2 to induce the expression of the adhesion molecule CD169/SIGLEC1 in myeloid lineage as an early marker of viral infection was demonstrated through flow cytometry^[87-89]^. In this context, we demonstrated that the high median fluorescence intensity ratio of CD169 between monocytes and lymphocytes (CD169 RMFI) in COVID-19 patients correlated with CD8 T-cell senescence and exhaustion (CCR7, CD45RA, CD28, CD27, CD57), and with B-cell maturation and differentiation markers (CD45, CD19, CD27, IgM, IgD). The SARS-CoV-2 spike protein has also been shown to activate CD169 RMFI in a dose-dependent manner^[90]^. Recently, the alteration of CD169 and HLA-DR expression in monocytes and inflammation indices in COVID-19 patients upon different waves of the pandemic and their persistence in PASC individuals were also observed^[91]^.

Furthermore, in the context of new biomarkers associated with COVID-19, the contribution of the endogenous human retrovirus W family envelope protein (HERV-W ENV) was evaluated. By flow cytometry analysis, the expression of HERV-W ENV was found elevated in leukocytes from COVID-19 patients but not in healthy donors, and the expression correlated with markers of T-cell differentiation and exhaustion, cytokine levels in the blood, and severity of pneumonia. Notably, HERV-W ENV expression reflected the respiratory outcome of COVID-19 patients during hospitalization^[92]^.

Although most infected people develop asymptomatic or mild COVID-19 disease, SARS-CoV-2 can induce profound immune dysregulation, leading to chronic inflammation.

Several works have demonstrated the alteration and persistence of inflammation and complex symptoms even after SARS-CoV-2 infection. PASC subjects may develop symptoms that persist or emerge beyond the acute phase of COVID-19, lasting for weeks or months^[93-95]^. Indeed, dysregulation of immune cell subtypes was found in convalescent COVID-19 patients up to 6 months after infection^[96,97]^, including alterations in mucosal immune parameters, redistribution of CD8+β7 Integrin+ T cells and IgA^[98]^. It was recently shown that the inflammatory state of PASC is due to ongoing neutrophil activation, alterations in B-cell memory, and self-reactivity more than one year after COVID-19^[99]^. The dysfunction of the immune system and the resulting inflammatory state can, therefore, be considered as prognostic markers for the outcome of the disease and for possible re-infection.

Further studies using flow cytometry also offered new treatment insights for PASC individuals with long-standing chronic inflammation. The administration of thymosin alpha 1 has been shown to be very effective in compromised PASC individuals, restoring an anti-inflammatory response in those who require respiratory support during the acute phase, and in those with systemic and psychiatric symptoms specific to PASC^[100]^.

In a recent study using “omics” analytical approaches combined with mass cytometry, immunophenotypic dysregulations in CD4 and CD8 T cells were highlighted in individuals showing post-SARS-CoV-2 infection alterations (LC). Moreover, an uncoordinated adaptive immune response to SARS-CoV-2 was demonstrated^[101]^.

In the cellular context, flow cytometry made it possible to characterize the harmful conditions of COVID-19, especially in the immunological context. At the same time, this initial evidence enriched the complex scenario found in PASC individuals, thus opening a possible way for research.

### EVs

In recent years, multiparametric flow cytometry technology has also been applied in the field of extracellular particle (EP), including EVs of animal and plant origin^[102]^.

Research groups are currently working to improve conditions for the EVs detection and evaluation by flow cytometry. A standardized protocol is crucial for characterizing EVs and defining their possible role as diagnostic markers.

To address these issues, a working group (WG) consisting of researchers in EV-flow cytometry (FC) from the International Society for Extracellular Vesicles (ISEV), the International Society for Advancement of Cytometry (ISAC), and the International Society on Thrombosis and Haemostasis, Inc. (ISTH) has developed a consensus list of essential information that should be provided about EV-FCs. This list is based on the MISEV and MIFlowCyt guidelines. All critical issues related to sample staining, detection and measurement of EVs, and experimental design in manuscripts reporting EV-FC data are analyzed here in a specific manner to EVs (MIFlowCyt-EV). MIFlowCyt-EV has been developed specifically for the reporting of single EV-FC experiments and is generally applicable to other small particles, such as viruses^[103]^. Considering the rapid evolution of analysis tools and methods, MIFlowCyt-EV provides a framework for sharing EV-FC results by not prescribing specific protocols and will be improved and revised periodically as EV-related technologies and applications develop, as is the case with MIFlowCyt and MISEV guidelines.

Key information regarding the flow cytometry approach in the detection and characterization of EVs has been brought together in a review aimed at promoting guidelines for robust and reproducible results^[103]^. Furthermore, in a recent work, a method based on flow cytometry imaging (IFCM) was proposed to characterize plasma EVs without previous isolation, in order to exclude possible biases caused by EV isolation^[104]^. Although there is ongoing debate over consensus on specific markers and protocols for EVs, the application of conventional flow cytometry assay in EV research could aid in the development of standardized methodologies. For example, regarding the general use of carboxyfluorescein diacetate succinimidyl ester (CFSE) in the characterization of EVs, it was observed that when cells were labeled with CFSE, there was a fluorescence transition on EVs that were reconstituted from the pellet but not on those that remained in the supernatant, suggesting the indirect labeling of EVs based on the mode of biogenesis as a specific method for distinguishing exosomes and ectosomes. Thus, this was a good approach to characterizing EVs by distinguishing the ectosomes produced from the outer cell membrane labeled by CFSE from the exosomes formed inside the cell by the multivesicular bodies not labeled by CFSE^[105]^.

Advances in flow cytometry have opened the possibility for analyzing EVs at high resolution, highlighting not only the different aspects that characterize a single EV, but also the complexity and different functions [Table 1]. In particular, to better analyze EVs less than 100 nm in size, such as exosomes, nano-flow cytometry methods are increasingly used in the field^[106]^.

**Table 1:** The contribution of flow cytometry in the study of EVs in the context of COVID-19 and PASC

**EVs**	**Sample**	**Disease**	**Isolation methodology**	**Detected molecules**	**Characterization methodology**	**Ref.**
Exosomes	Plasma	COVID-19	Centrifugation	Anti-SARS-CoV-2-S-RBD	Anti-CD63-conjugated bead purification	[56]
Large EVs	Plasma	COVID-19	Centrifugation	E-cadherin (CD144+), PECAM-1 (CD31+CD41-), E-selectin (CD62E+)	Gigamix (beads of sizes 0.1, 0.16, 0.2, 0.24, 0.3, 0.5, and 0.9 μm)	[111]
Small and Large EVs	Plasma	COVID-19	Ultracentrifugation and filtration (Small EVs) Centrifugation and filtration (Large EVs)	CD63, CD81, CD41, CD235a, CD31, CD62P	Sub-micron particle 0.1, 0.2, 0.5 µm	[116]
Small and Large EVs	Serum	COVID-19	Capture beads coated with antibodies (CD9, CD63, CD81)	TF (CD142) and CD63	Capture beads coated with antibodies (CD9, CD63, CD81)	[118]
Small and Large EVs	Serum	COVID-19	Ultracentrifugation	CD49e, CD209, CD86, CD133/1, CD69, CD142, CD20	Capture beads coated with antibodies (CD9, CD63, CD81)	[119]
Small and Large EVs	Plasma	COVID-19		CD62E, TF, ACE2, CD62P, CD45, PDGF-β, anti-SARS-CoV-2-NP	Gigamix (beads of sizes 0.1, 0.16, 0.2, 0.24, 0.3, 0.5, and 0.9 μm) and calcein-AM	[120]
Small and Large EVs	Plasma	COVID-19	Centrifugation	CD253, CD144, CD62E, CD41, CD62P, CD142, CD141, EPCR, CD4, CD8, CD28, CD154, CD14, CD22	Megamix beads (0.5, 0.9, 3, and 0.78 μm)	[121]
Small and Large EVs	Plasma CSF	COVID-19	Centrifugation	Lactadherin, CD61, C3a, MPO, C4d, TCC, CD51/61, CD45, CD142 Lactadherin, CD51/61, CD61, TCC, MPO, CD45	Megamix-plus SSC beads of sizes 0.16, 0.20, 0.24, and 0.5 μm	[122]
Small and Large EVs	Cells culture supernatant Plasma	COVID-19	Centrifugation and ultracentrifugation	ACE2, CD81, CD63, TSG101	Fluorescent polystyrene beads (180, 240, 300, 590, 880, and 1,300 nm)	[67]
Small and Large EVs	HEK293A supernatant Serum	COVID-19	Filtration, centrifugation and ultracentrifugation	SARS‐CoV‐2 Spike S1, CD63, CD45, CD38+, IgA+, IgG+	Silica nanoparticles (66, 91, 113, and 155 nm)	[123]
Small and Large EVs	Plasma	COVID-19 LC-19	Centrifugation Anti-CD63-conjugated bead purification	CD14, CD1C, CD11C, CD142, CD105, MCSP, CD4, CD8, CD41b, CD62P	Capture beads coated with antibodies (CD9, CD81)	[130]
Small and Large EVs	Plasma	COVID-19	Filtration, centrifugation and ultracentrifugation	CD41, CD14, CD66, CD146, TF, TFPI	CD81, CD9, CD63, and calcein-AM	[117]
Small and Large EVs	Plasma	COVID-19	Centrifugation and polymer for gently precipitates	CD31, CD45, N‐cadherin, CD56, CD140b, CD34 and CD42b, CD63	Fluorescent beads (200-500-1,000 nm) and CFSE	[125]
Small and Large EVs	293F cell media carrying the SARS-CoV-2 spike protein	COVID-19	Centrifugation, FPLC-mediated size exclusion, ultrafiltration	CD29, CD326, CD56, HLA-DR, CD3, CD29, CD44, CD24, CD146, MCSP, ROR1, CD49a	Capture beads coated with antibodies (CD9, CD63, CD81), CFSE	[126]

EVs: Extracellular vesicles; PASC: post-acute sequelae of SARS-CoV-2 infection; TF: tissue factor; NP: nucleoprotein; CSF: cerebrospinal fluid; SSC: Side Scatter; LC: Long COVID; CFSE: carboxyfluorescein diacetate succinimidyl ester; FPLC: fast performance liquid chromatography.

Results obtained by other methods^[55,56]^ showed a possible contribution of EVs in the persistence of symptoms, considering their ability to spread viral material and the inflammatory response.

A more detailed characterization of EVs is essential to define their contribution not only in physiological conditions, but especially in various diseases. Numerous studies have examined the potential involvement of EVs in different tumor-related, inflammatory and immune processes^[107]^.

The study of EVs has been intensified in the cardiovascular field to define their pathophysiological importance. Davidson *et al.* summarize the main separation methods and techniques for identifying and determining EV proteins, RNAs, and lipids content, including methodologies for the clinical use of EVs in cardiovascular diseases^[108]^.

In the context of COVID-19, several detrimental conditions, effects related to coagulation alteration, thrombotic complications, and endothelial activation have been widely described^[109,110]^. The plasmatic EVs expressing endothelial markers such as CD62E-selectin (CD62E) were characterized by flow cytometry. The increase in endothelial CD62E+ EVs in COVID-19 patients at the time of hospitalization was significantly associated with critical disease, highlighting the potential role of circulating CD62E+ EVs as a marker to identify individuals with increased risk of fatal outcomes^[111]^.

The role of activated platelets that can release EVs as bioactive shuttles, which transfer their contents, has been widely discussed. In particular, in the case of viral infection, platelets are activated and release EVs that also contain virions^[112,113]^. Indeed, in SARS-CoV-2 infected cells, tissue factor activates thrombin, which signals to platelets of protease-activated receptors^[114]^.

Recent research demonstrated that the mean fluorescence intensity of the CD62P-selectin (CD62P) platelet activation marker was significantly higher in the large EVs of COVID-19 patients compared to healthy donors. The characterization of large EVs with cell-derived markers showed higher expression of CD41 (platelets), CD235a (erythrocytes), and CD31 (endothelial cells) than in healthy donors^[115,116]^.

Brambilla *et al.* demonstrated that in patients with SARS-CoV-2 infection, large plasma vesicles show a procoagulant potential dependent on the presence of tissue factor. This study showed that in patients, the resolution of the disease led to the restoration of physiological concentrations and procoagulant function of large vesicles^[117]^.

The high expression of tissue factor (also known as CD142) on the surface of vesicles of COVID-19 patients was also found to be biologically active and a reliable prognostic marker correlating with serum levels of TNF-α^[118,119]^. An increase in EVs from cells involved in COVID-19-associated coagulopathy, particularly those derived from activated pericytes expressing SARS-CoV-2-nucleoprotein (NP), was also found. This study showed that after discharge, EVs of endothelium decreased, while those of leukocytes and platelets increased further. This suggests that in the disease course, there is a differential distribution of EVs of various cellular origins, contributing to sustained activation following the acute phase^[120]^.

Supporting coagulation alterations and thrombotic events, EVs of patients with severe COVID-19 showed elevated levels of platelet markers (CD41) and coagulation factors. Patients with moderate/severe disease showed EVs expressing significantly higher levels of immune cell markers (CD4/CD8/CD14)^[121]^.

Interestingly, EVs were assessed by flow cytometry in plasma and CSF. High levels of circulating EVs in plasma derived from neutrophils (MPO+) and platelets (CD61+) and complement component C5b-9 (TCC+) were observed in patients with COVID-19 compared with controls^[122]^.

An increase in plasma-level circulating EVs expressing ACE2 (evACE2) capable of neutralizing SARS-CoV-2 infection by competing with cellular ACE2, supporting an antiviral mechanism, was also demonstrated in COVID-19 patients. The authors suggest a mechanism in which ACE2-expressing small EVs can delay the binding of cellular ACE2 by linking SARS-CoV-2. This is due to the higher affinity of ACE2 proteins present on EVs to the virus spike protein^[67]^.

Interestingly, using nano-flow cytometry, the serum EVs from COVID-19 patients were analyzed and multiparametric phenotyping was performed to characterize immune-associated subpopulations of EVs. The presence of EVs derived from specialized small immune cells (classical monocytes, B cells, NK cells) reflects the dynamics of COVID-19 progression and partial recovery of the immune system toward the final stage of infection^[123]^.

In another study, it was shown that plasma exosomes of COVID-19 patients contain double-stranded RNA (dsRNA) of SARS-CoV-2 and are responsible for the production of IL-6, IL-8, and TNF-α by mononuclear cells, particularly in CD4+ T cells, CD8+ T cells, and CD14+ monocytes^[124]^.

With respect to the severity of COVID-19, CFSE staining was used to identify the cytoplasm of EVs, and CFSE-labeled EVs were stained with antibodies specific for the antigens of interest to determine the immunophenotype of each EV subgroup^[125]^. In addition, delivery of Spike EVs to PBMCs also characterized by CFSE resulted in specific immune activation as assessed by expression of T-cell activation markers. Furthermore, Spike EVs were largely taken up by antigen-presenting cells (monocytes, dendritic cells, and B cells)^[126]^. All these aspects confirm how CFSE tagging can contribute to the characterization of these EVs in the course of COVID-19 disease and its associated etiopathogenetic mechanisms.

Considering their ability to convey pro-inflammatory and immunostimulatory information to different parts of the body, these data support the involvement of EVs in the disease.

The severe multiorgan failure and the inflammation resulting from COVID-19 progression persist over time after infection, leading to PASC syndrome, often associated with wide-ranging neurological disorders^[127-129]^. It is essential to characterize EVs in individuals with sequelae post COVID-19 using flow cytometry. In a recent paper, the profiles of EVs remain distinct several weeks after COVID-19 disease. Co-culture with EVs has an impact on the effector functions of healthy T cells and their metabolism *in vitro*, demonstrating suppressive capacity^[130]^.

## DISCUSSION AND CONCLUSION

COVID-19 can be defined as a multidistrict disease because, despite the primary sites of infection (nasopharynx, trachea, and nasal olfactory mucosa)^[131]^, the SARS-CoV-2 virus is able to reach different tissues and infect several cells^[24]^. The cytopathic effect induced by SARS-CoV-2, in particular alteration of the endoplasmic reticulum, the Golgi apparatus, and the cytoskeleton, has been widely documented^[36,38-40]^. These impairments compromise cellular activity and also interfere with the biogenesis and secretion of EVs such as exosomes and MVs.

EVs are fundamental in cellular communication through both the expression of specific surface markers and the release of their contents^[15,132]^. Their phenotype and internal materials reflect the state of the organism, where EVs could restore disrupted homeostasis in diseases or promote viral propagation during infections^[133]^. Due to their size and surface markers heterogeneity, EVs have been widely studied to establish guidelines for their characterization. Several methodological approaches have been explored to standardize protocols^[5,55,134]^, but the study and characterization of EVs are ongoing. Current guidelines are contained in the MISEV 2024 document^[5]^. Additionally, efforts to establish standardized protocols and identify specific EV markers are evolving. For example, the EV-FC working group, comprised of researchers from ISEV, ISAC and ISTH, has developed a consensus framework outlining the minimum information required for EV-FC studies.

In the context of COVID-19, various studies have highlighted the potential role of EVs as markers of pathology. Specifically, EVs have shown both pro-SARS-CoV-2 and anti-SARS-CoV-2 properties. Research has demonstrated that exosomes isolated from the plasma of COVID-19 patients can carry the Spike protein, RNA residues, and SARS-CoV-2 viral particles. These exosomes may act as decoys to neutralize antibodies^[56-59]^. Additionally, ACE2-expressing plasma EVs have been shown to block the spread of SARS-CoV-2^[67]^.

Compared to other methods, the multiparametric approach of flow cytometry has been fundamental to identify immunologic impairment and evaluate new predictive biomarkers in COVID-19 patients^[90-93]^. Advances in flow cytometry technology have enabled the adaptation of this approach to the study of EVs.

Furthermore, subpopulations of immune-associated EVs in the sera of COVID-19 patients have been characterized. Notably, nano-flow cytometry has allowed for the identification of EVs derived from specialized immune cells (monocytes, B cells, and NK cells), which reflect the progression of COVID-19. In this context, the ability of EVs to promote immune response has also been demonstrated^[123]^. These findings highlight the important role of EVs in COVID-19, particularly in relation to disease severity and complications.

A notable finding is the close relationship between EVs and cells. The production of EVs with specific markers, such as those from coagulation or immune system cells, is closely dependent on the cell of origin and reflects the status of different tissues. This connection further supports the use of circulating EVs as indicators of changes in different body districts.

COVID-19 is known to cause multiorgan dysfunction, particularly neurological changes that can persist even after the infection has resolved. Several studies have characterized plasmatic EVs of individuals with LC who experience sequelae after infection^[71-72]^. For example, neuron-derived EVs isolated from the plasma of PASC individuals have been characterized using various methods such as chemiluminescent microparticle immunoassay, electron microscopy, Luminex bead assay, and ELISA. These studies highlighted the capacity of these EVs to carry SARS-CoV-2 proteins and their role in neuronal dysfunction^[57]^. Moreover, in COVID-19 patients, EVs have been shown to deliver fragments, RNA and virions of SARS-CoV-2. In PASC, this evidence highlights the role of EVs as a vehicle for the virus, thus supporting the Trojan horse theory^[135]^. In this way, surveillance of the immune system is deviated, since transported proteins act as bait for neutralizing antibodies, while internalization of the proteins and virus promotes spread to different districts.

There is a need for new evidence from flow cytometry to characterize tissue-derived plasmatic EVs in COVID-19 and, more specifically, in the post-infection context. Plasma and serum, components of blood, are the only fluids circulating throughout all organs and carrying different materials including cytokines from different tissues, proteins, antibodies, electrolytes, and EVs of different cellular origins. While EVs are normally present in plasma, it is crucial to characterize them based on structural markers (CD63, CD81, CD9) and cell-derived markers to define threshold values for both concentration and marker expression.

To accurately characterize EVs, it is important to combine flow cytometry with other methods, such as “omics”^[136]^. This approach can also be applied to other diseases, such as post-COVID-19, to identify new biomarkers, including EVs.

Once physiological parameters are defined, it will be possible to identify specific markers associated with different alterations.

**Figure 3:**
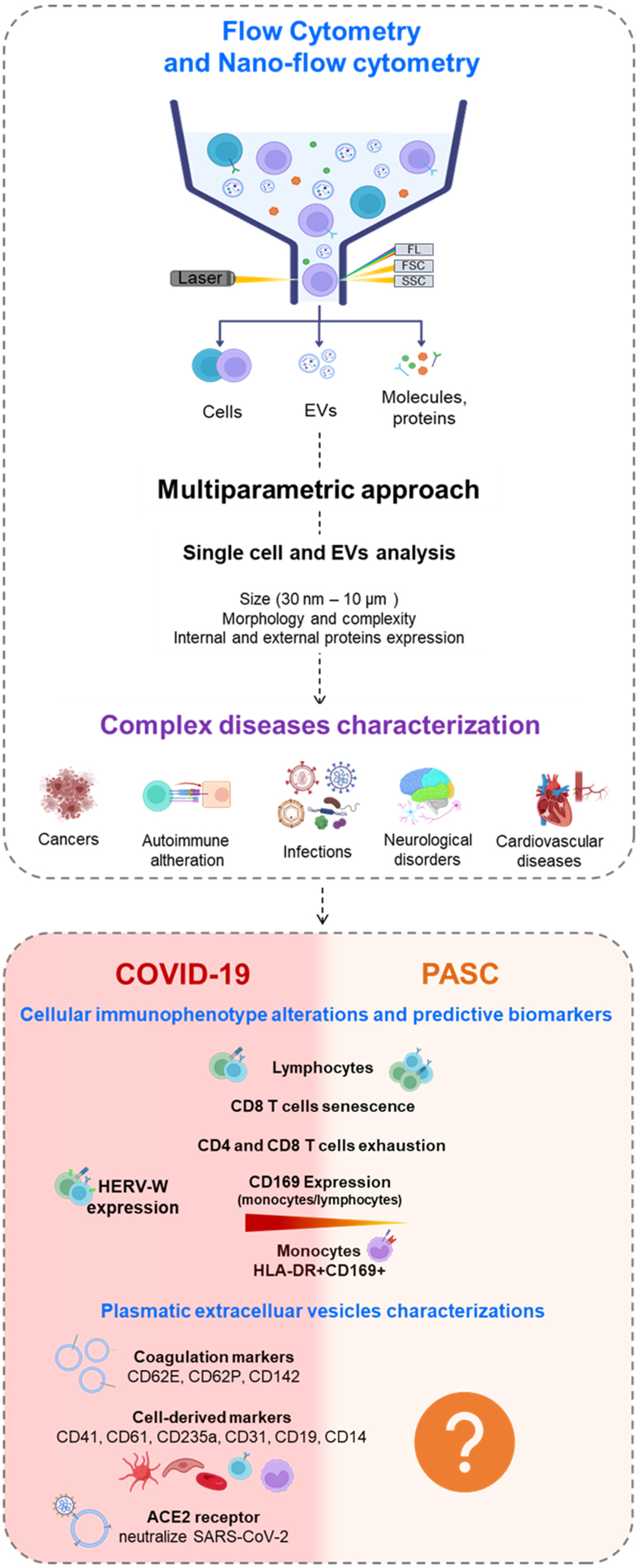
The employment of flow cytometry and nano-flow cytometry in COVID-19 and PASC syndrome. Created with BioRender.com. PASC: Post-acute sequelae of SARS-CoV-2 infection.

Flow cytometry is a multiparametric approach that simultaneously evaluates the size, content, quantity, and expression of both internal and external markers. Advances in nanotechnology now enable the analysis and sorting of small-diameter particles such as viruses, bacteria and EVs^[137,138]^, aided by the development of nanoscale flow cytometers [Figure 3].

Flow cytometry is a multiparametric approach to analyze material of different sizes (cells, EVs, molecules, and proteins). Physical feature-dependent signals (Forward Scatter or FCS and Side Scatter or SSC) and the presence of fluorescent markers (fluorescence or FL) are generated from a light released by a laser. Due to this ability, it is widely used to study various diseases.

## DECLARATIONS

### Copyright

© The Author(s) 2024.
